# Complementary and Alternative Medicines for the Treatment of Hepatitis C: Perspectives of Users and CAM Practitioners

**DOI:** 10.1155/2020/3932690

**Published:** 2020-12-15

**Authors:** Salamat Ali, Shahan Ullah, Vibhu Paudyal, Mashhood Ali, Muhammad Khalid Tipu, Tofeeq Ur-Rehman

**Affiliations:** ^1^Department of Pharmacy, Quaid-i-Azam University, Islamabad, Pakistan; ^2^School of Pharmacy, University of Birmingham, Birmingham, UK; ^3^Department of Gastroenterology, Pakistan Institute of Medical Sciences, Islamabad, Pakistan

## Abstract

**Background:**

Despite substantial progress in the treatment of hepatitis C through the use of direct-acting antivirals which have been shown to cure the disease, complementary and alternative medicines (CAM) are popular among patients as a substitute or complement of allopathic medicines. This study aimed to explore the perspectives of patients and CAM practitioners on the use of CAM for the treatment of hepatitis C in Pakistan.

**Methods:**

A cross-sectional design was adopted. Participants (CAM practitioners and patients) were recruited from the capital and two provinces: Khyber Pakhtunkhwa and Punjab of Pakistan. A survey using paper-based questionnaires, each specific for patients and CAM practitioners, was conducted to gather information pertaining to demography, disease status, treatment history, and participants' perspectives (about the disease, reasons to switch to CAM, and referring source).

**Results:**

A total of 417 respondents (*n* = 284 patients, *n* = 133 practitioners) were recruited. Of the total patients, 170 (59.9%) had started CAM during the previous three months. There were 168 (59.2%) of the total patients who had used allopathic treatments for hepatitis C prior to their use of CAM. The confidence in CAM (24.6%), high cost (19%), and unbearable side effects (52.1%) of allopathic medicines were the main reasons to switch to CAM treatment. Majority (49.3%) of the patients were referred to CAM on the recommendations of relatives or care givers (17.3%) whereas only 9.5% were referred by health care professionals. Out of 133 practitioners, 48 (36.1%) were practicing herbal medicines. From practitioners' perspectives, club-moss (*Lycopodium clavatum)* was the best treatment option for hepatitis C. The majority, 73 (54.9%), of the patients had chosen to use CAM because of the side effects of allopathic medicines. Patients who had previous “good experience” with CAM were the most common referral source (56.4%) for CAM use in hepatitis C.

**Conclusions:**

Patients' beliefs in CAM, side effects of allopathic therapy, high cost of allopathic medicines, and referrals from previous CAM users are key factors in the switching of hepatitis C patients to CAM.

## 1. Introduction

Approximately, 71 million people are infected with hepatitis C virus (HCV) worldwide [1]. Pakistan in the South Asian region has a high burden (6.0%) of active HCV infection [2]. The new direct‐acting antiviral agents (DAAs) have changed HCV treatment landscape leading to cure rates of over 90% [3]. Furthermore, the pan-genotypic DAAs have shown promising results (>95%) in complicated patients and have reduced the pill burden [4]. Nevertheless, during the last few years, an increasing number of reports suggest the availability of complementary and alternative medicines (CAM) as a natural remedy in the use of HCV treatment [5]. However, user and CAM provider perspectives in the use of CAM in the treatment of HCV have not been explored before. The term CAM is used collectively for all those remedies that are not included in the western allopathic system [6]. National Center for Complementary and Alternative Medicine (NCCAM) defines CAM as a group of diverse medical and health care systems, practices, and products that are not presently considered to be part of conventional medicine [7]. CAM uses natural health products such as vitamins, homeopathic remedies, herbal medicines, spiritual healing, chiropractic remedies, and prayers [8].

For practicing CAM, the regulation (production and safe use) of herbal medicines is directly governed by World Health Organization (WHO) [9–12]. Being indigenous and of easy access, CAM is very popular in Asia specifically in India and Pakistan and it is widely recognized that CAM resources have demonstrated to be a supplement to allopathic medicines [13, 14]. Practicing and education of the CAM are regulated by the Ministry of National Health Services Regulation and Coordination Islamabad (NHSR&C), through the National Council for Tibb (Eastern medicines) [15]. Drug Regulatory Authority of Pakistan (DRAP) started the proper enlisting of CAM in Pakistan and provisional list of enlisted CAM is available on DRAP official website [16].

From studies in other clinical areas, the predictors of CAM use often included the referrals, availability of information on the Internet, high cost of allopathic medicines, patient's interest in deciding his/her medical treatment, and lack of beliefs in allopathic medicine [17–19]. The migrants in developed countries continue to use their traditional remedies based on their beliefs during their stay in these countries [20]. Also, CAM has been utilized by the general public who have faith in homeopaths, hakeems, and spiritual healers [21–23]. However, literature around exploring the perspectives of user and provider associated with CAM use in hepatitis C is limited.

This study aimed to explore the perspectives of patients and CAM practitioners on the use of CAM for the treatment of hepatitis C in Pakistan.

## 2. Materials and Methods

A cross-sectional study design was adopted. Participants were recruited from twelve districts with a high prevalence of hepatitis C of the capital and two provinces, i.e., Khyber Pakhtunkhwa and Punjab, Pakistan, between May 2016 and February 2018.

### 2.1. Settings and Participants

CAM practitioners were randomly selected from the latest list provided by the local health department and were invited to participate through telephone or on-site requested interview. Only those practitioners registered with their respective national council were recruited for the study. Patients visiting the private clinics of CAM during researchers' visits were approached for the study. First five patients from each clinic on the day of interview who were polymerase chain reaction- (PCR-) positive were selected. Patients of 18 years or above, diagnosed with hepatitis C, and using CAM were included. Patients who refused to be interviewed were excluded and the equal numbers were of patients taking new appointments.

The perspectives of patients and practitioners of CAM in their use of CAM for the treatment of hepatitis C were explored considering the characteristic variables: the demography, disease status, perception about disease and diagnosis, referring source, and the reasons for leaning to CAM.

### 2.2. Data Collection Tool and Its Administration

Two questionnaires, each specific for patients and CAM practitioners, were developed and were moderated by a team of experts, which comprised of a hepatologist, a pharmacist, a homeopathy, and an herbalist. The final versions of questionnaires were similar to the other published studies of CAM use [24, 25]. Prior to data collection, the survey tools were pretested with 15 practitioners of CAM and 30 patients using CAM who attended/have clinics in vicinity (as a pilot conduct). Face-to-face administration of questionnaires was undertaken by the researchers. The data collection was carried out by a team comprising a researcher and five trained postgraduate pharmacists who independently surveyed the allotted districts. Questionnaires missing substantial information were excluded.

The questionnaires used common and validated components to explore patients' and practitioner' perspectives. The first part of questionnaire exploring patients' perspectives included descriptive questions to acquire information pertaining to demography, socioeconomic status (medicine affordability), disease status (known to his disease since), disease symptoms, treatment history (used modern medicines or CAM in the past), diagnosis, and mode of acquiring infection (blood transfusion, razor cuts/abrasions, intravenous drug use (IVDU), sexual contact, and unsterilized syringes). The second part included three components exploring patients' perceptions about the CAM, referring source, and the reasons for leaning to CAM. The third part of questionnaire contained queries pertaining to patients' past experience of using allopathic medicines (access, side effects, and availability of services around counselling of medication use) and reasons for discontinuation of allopathic medicines.

The questionnaire exploring practitioners' perspectives contained general queries (pertaining to practitioners' qualification, speciality, registration with the CAM council, and professional experience). The second part of questionnaire inquired descriptive queries pertaining to perception about CAM and the best CAM regimen available for hepatitis C. Third part of the questionnaire included the components to explore the practitioners' observations pertaining to referring source and the reasons for leaning to CAM. There may be a researcher bias and patient may have not been telling the truth out of fear. All participants were communicated directly, study purpose was explained, and any query was resolved by further elaboration to minimize this bias. We selected twelve districts from two provinces that have high prevalence of hepatitis C and recruited 30 patients and 15 CAM practitioners from each district as the study sample. The study was approved by the Bioethics Committee at Quaid-i-Azam University, Islamabad, Pakistan (DFBS/248). A written consent was sought from the participants prior to start of interview.

### 2.3. Statistical Analysis

The data were anonymized, coded, and then analysed using IBM SPSS Statistics data editor (V.24). All questionnaires that contained missing values or incomplete information were excluded from the final analysis. Frequency distribution was determined for the demographic variables, diagnostic parameters, and exposures to treatments. Chi-square test was performed to explore any relationship between referral source and gender, referral source and practitioner, reasons for patients to switch to CAM and gender, and reasons for patients to switch to CAM and practitioner. Kruskal–Wallis test was performed to rank the difference of factors such as referrals and reasons to shift to CAM. Any difference between the practitioners' and patients' responses pertaining to referrals and reason to use CAM was compared using paired sample *t*-test. A *P* value of less than 0.05 was taken as significant.

## 3. Results

### 3.1. From Practitioners' Perspective

**Figure 1:**
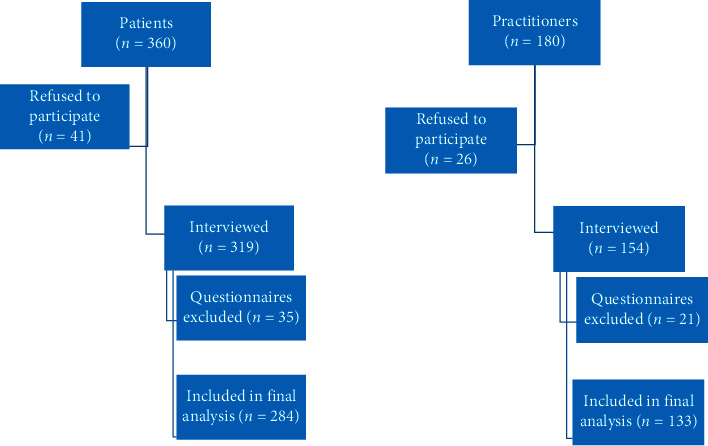
A flow chart showing the details of participants and final analysis.

A total of 540 participants involving 360 patients (30 from each district) and 180 practitioners (15 from each district) were targeted for interview, of whom 41 patients and 26 practitioners refused to participate. Thirty-five questionnaires of patients and 21 questionnaires of practitioners were excluded from the analysis due to insufficient information or incomplete data. A total of 417 questionnaires were found appropriate for the final analysis including 284 (68.1%) patients and 133 (31.9%) practitioners. A flow chart in Figure 1 shows this in more detai.

#### 3.1.1. Characteristics of Respondents

Out of 284 patients interviewed in this study, the majority, 149 (52.5%), were aged above 60 years, and 173 (60.9%) were females. Socioeconomically, 84 (29.6%) patients were dependent on parents and 69 (24.3%) on spouse for the cost of their anti-HCV treatment. There were 89 (31.3%) patients who had acquired HCV infection via razor cuts or abrasions (Table 1).

**Figure 2:**
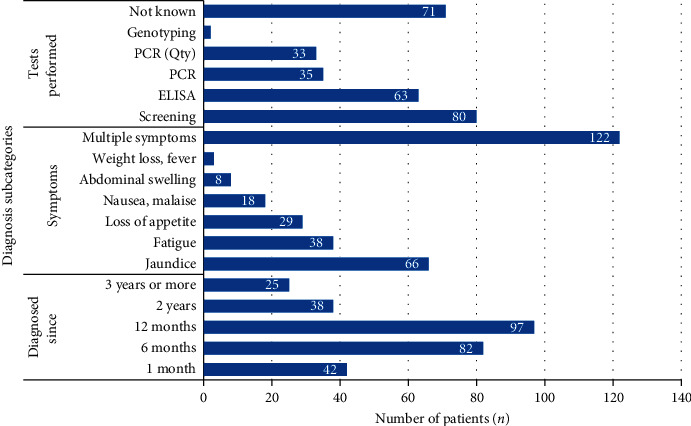
Patients' response regarding symptoms and diagnosis of hepatitis C. Abbreviations: PCR = polymerase chain reaction; Qty = quantitative; ELISA = enzyme-linked immunosorbent assay.

Ninety-seven (34.2%) of the patients were aware of their HCV infection for at least one year. During interview, the symptoms reported by patients included jaundice, 66 (23.2%); fatigue, 38 (13.4%); and nausea, 18 (6.3%). A substantial number, 122 (43.0%), of patients had multiple symptoms of the disease (more than one). Eighty (28.2%) patients had gone through HCV screening test prior to starting CAM (Figure 2).

Out of 133 practitioners interviewed in the study, 48 (36.1%) practitioners were practicing herbal medicines, while 45 (33.8%) were practicing homeopathic medicines. There were 49 (36.8%) practitioners who had higher secondary level education before the CAM course. Seventy-five (56.4%) practitioners had more than 10 years of experience in CAM (Table 1).

#### 3.1.2. Treatment Choices and Adjunct Alternative Medicines

**Figure 3:**
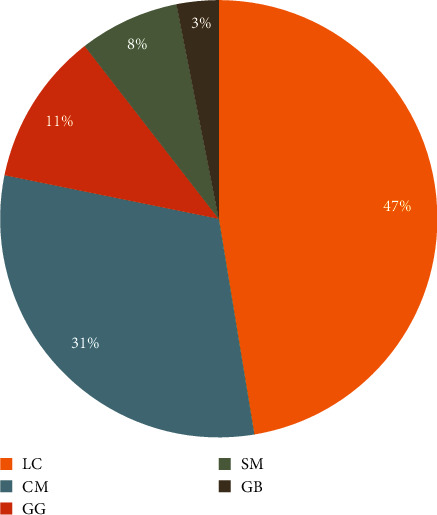
Treatment choices and adjunct alternative medicines as reported by CAM practitioners. LC = *Lycopodium clavatum, CM* = *Cardus marianus, GG* = *Glycyrrhiza glabra*, SM = *Silybum marianum*, and GB = *Ginkgo biloba*.

While asking about the best choice of treatment, 63 (47.4%) of practitioners believed club-moss (*Lycopodium clavatum)* to be the best treatment option for hepatitis C, followed by 41 (30.8%) silybum (*Cardus marianus)*, and 15 (11.3%) licorice root (*Glycyrrhiza glabra*). Few of the practitioners, 10 (7.4%), were of the opinion to use milk thistle (*Silybum marianum*) and 4 (3.1%) considered maidenhair tree (*Ginkgo biloba*) as hepatoprotective and as an adjunct therapy, respectively (Figure 3).

#### 3.1.3. Patients' Perspectives on the Use of CAM

Of the total patients who visited the clinics, 104 (36.6%) reported that they had used anti-HCV medicines for more than five months and a substantial number, 170 (59.9%), of the patients had started CAM during previous 3 months (Table 1).

Of the self-reported prior treatments, 122 (42.9%) patients reported that they had used injection based treatment (pegylated-interferon) prior to switching to CAM and 46 (16.2%) had used tablet based treatment (sofosbuvir). There were 47 (16.6%) patients who had used hepatoprotective agents like silymarin and 69 (24.3%) had medicines for symptomatic relief for day-to-day illness. The details are available in Table 2.

#### 3.1.4. Reasons for Their Use of CAM

Among the reasons for patients to switch to CAM, majority, 148 (52.1%), of the patients reported that they had switched to CAM due to unbearable side effects of allopathic medicines. A total of 70 (24.6%) patients had confidence in CAM. There was no significant difference between genders regarding reasons for use of CAM; *χ*^2^ (4, *N* = 284) = 2.52, *P* > 0.05. Furthermore, most of the side effects, which led to the discontinuation of allopathic medicines, were injection based treatments (pegylated-interferon; PEG-INF) and out of those, 36 (29.5%) patients complained of having multiple side effects (fever, anemia, and depression) and 43 (35.2%) patients reported nausea/vomiting from injection based treatments. Of the total, 54 (19.0%) patients reported that the allopathic medicines were expensive and they were unable to make out-of-pocket (OOP) purchase (Table 2).

#### 3.1.5. Source of Referral for CAM

A substantial number, 160 (56.3%), of the patients were referred to CAM use by the relatives, followed by allied health professionals (technicians), 53 (18.7%), and care givers, 49 (17.2%).

Few, 12 (4.2%), of the patients indicated that they shifted to CAM while going through print media or advertisement brochures of the provider. There was no significant difference among patients regarding referrals for CAM; *χ*^2^ (4, *N* = 284) = 10.71, *P* > 0.05. The details are given in Table 3.

**Figure 4:**
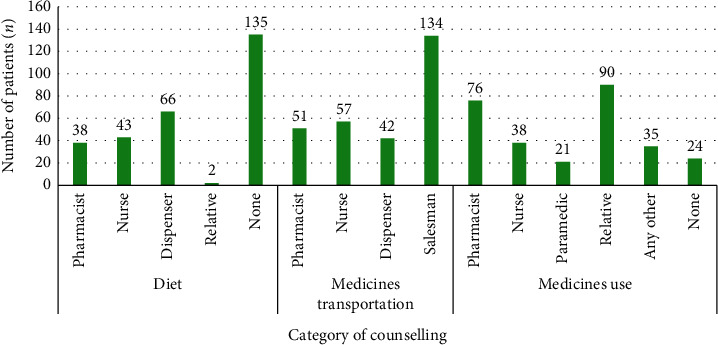
Patients' response regarding provision of counselling pertaining to allopathic medicines.

A total of 260 (91.5%) patients reported obtaining any form of counselling on their allopathic medicines during the dispensing of medicines, including 76 (26.8%) of the patients receiving counselling by the pharmacists, followed by nurses 38 (13.4%) and pharmacy technicians 21 (7.4%). A total of 24 (8.4%) patients reported not receiving any counselling. Plurality of the patients, 90 (31.7%), mentioned discussing their medicines use with relatives or care givers (Figure 4).

### 3.2. From Practitioners' Perspectives

Out of 133 practitioners, 48 (36.1%) had managed 21–50 HCV patients while 15 (11.3%) practitioners managed over 500 patients during their career (Table 1).

**Figure 5:**
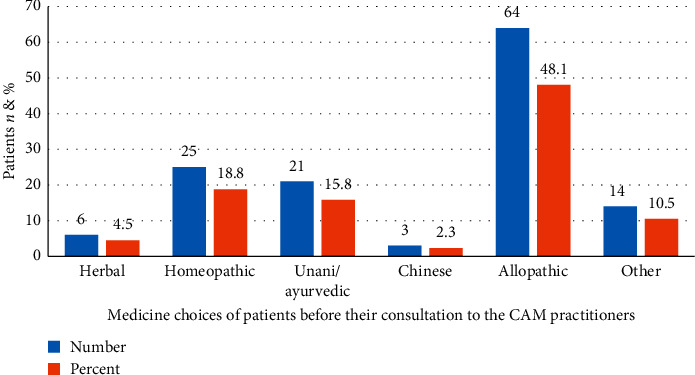
Medicine choices of patients before their consultation to the CAM practitioners.

According to practitioners' observations, there were 64 (48.1%) of their patients who had ever used allopathic medicines before their visit/consultation to the CAM practitioners. Moreover, few of the patients, 25 (18.8%), had a history of using homeopathic medicines while only 3 (2.3%) patients had been using Chinese remedies (Figure 5).

#### 3.2.1. Reasons to Switch to CAM

From the CAM practitioner perspectives, the reasons for shifting to CAM included side effects (73 (54.9%)), followed by confidence in CAM (23 (17.3%)) and cost of allopathic medicines (22 (16.5%)) (Table 3). Over 40% of the practitioners reported observing multiple side effects of allopathic medicines in patients (*n* = 58, 43.6%) followed by nausea and vomiting (*n* = 42, 31.6%) (Table 3). Among the complications from allopathic medicines, 43 (35.2%) patients had history of low blood cells counts from injection based treatments. There were multiple complications observed in 32 (26.2%) patients featured in the laboratory reports of patients (Table 3). A significant difference was observed among practitioners' response regarding reasons to switch to CAM; *χ*^2^ (4, *N* = 133) = 10.74, *P* < 0.05. When comparing the response pertaining to reason to switch to CAM between patients and practitioners, there was insignificant difference in the scores for patients' response (*M* = 56.80, SD = 56.36) and practitioners' response (*M* = 26.4, SD = 25.7) conditions; *t* (9) = 1.79, *P*=0.106^″^ (Table 3).

#### 3.2.2. Source of Referral for CAM

The most common referral source to the use of CAM as described by the CAM practitioners was their formerly treated patients (*n* = 75, 56.4%), followed by the patient's relatives (25 (18.8%)) and print media (18 (13.5%)).

A total of 26 (54.2%) of the herbal practitioners reported that their patients were referred by previously treated patients. According to the homeopathic practitioners, the common referral source to CAM was previously treated patients (32 (71.1%)), followed by relatives (8 (17.8%)) and print media (2 (4.4%)). Table 3 shows the details of observations of all practitioners regarding referring source and motives in their patients. There was a significant difference among practitioners regarding referrals to CAM; *χ*^2^ (4, *N* = 133) = 11.08, *P* < 0.05. Also, there was a significant difference in the scores for patients' response (*M* = 6.25, SD = 2.15) and practitioners' response (*M* = 4.39, SD = 1.15) conditions; *t* (132) = 8.53, *P*=0.000^″^ pertaining to referral source (Table 3).

## 4. Discussion

This study explored the perspectives of patients and practitioners of CAM in the treatment of hepatitis C in Pakistan. The results provide an evidence that a significant proportion (59.9%) of the patients had started CAM use alongside or as an alternative to anti-HCV treatment and the majority (52.1%) had confidence in CAM. The results demonstrated existence of common norm of transferring medical information by word-of-mouth in our society dominantly associated with high referrals by relatives and beliefs of patients in alternative medicines conforming earlier studies [19]. The health-seeking behaviour of the general public, especially in a developing country like Pakistan, demands bringing all CAM practitioners into the mainstream to serve as a contributor in health service provision and back-up for referral [21].

The socioeconomic status (as being dependent on parents or spouse), gender (more in female patients), age (60 years or above), and weight 61–80 kg were significantly associated with CAM use. These results are conformational to other studies [26–29] and depict the poor economic status of patients and referral existing norm in the society specifically in females and senior citizens.

The most common plants having antiviral activity, as reported by the practitioners, were club-moss (*Lycopodium clavatum,* 47.4%), silybum (*Cardus marianus*, 30.8%), and licorice root (*Glycyrrhiza glabra*, 11.3%). The scientific evidence of these plants or their combinations, described by the respondents of this study, was not substantiated for their use in HCV treatment and hence, there is still a risk that patients' treatment may be suboptimal [30]. CAM could be a significant contributor to the available treatment regimens against HCV, if sufficient evidence of indigenous plants is explored for their effectiveness. However, the benefits of CAM for curing hepatitis C and their lack of side effects cannot be ignored by the health care community suitably serving the users who had switched to CAM due to unbearable side effects of allopathic medicines.

Relatives, care givers, and allied health professionals were found to be the main referral sources to CAM for hepatitis C patients [17, 18]. Conversely to the previous studies, wherein it has been reported that health professionals do not influence the shift to CAM, we found that a small percentage of allied health professionals (technicians) do refer the patients to CAM practices. Moreover, the side effects like anemia and depression more likely influence the shift of patients to CAM [31, 32].

From practitioner's perspectives, the common referral source to CAM was the patient population, who benefited from alternative medicines. Additionally, the high cost of allopathic medicines is another key reason for the patients' leaning to CAM. The high cost of newer DAAs for hepatitis C treatment presents a considerable barrier in access to hepatitis C therapy for low-income patients in lower-middle-income countries (LMICs) because most of the users were dependent on their guardians.

In our study, fewer (4.2%) patients indicated that they shifted to CAM while going through print media or advertisement brochures of the provider. This finding, favourably, triggers the need for active participation of print media in terms of education of care givers of the patients regarding the availability of effective and safer treatments [33], which may prove helpful in improving patients' perspectives as the lack of awareness and counselling about medicines usage has been witnessed by both the users and providers.

This is the first multicentre study that aimed to explore the perspectives of patients and practitioners of CAM in the treatment of hepatitis C in Pakistan with an adequate sample population. The direct administration of the questionnaires by the principal investigator and by the trained pharmacists who surveyed in person improved the response rate. Moreover, we selected those districts which had a high rate of reported hepatitis C prevalence.

Like most of the studies, our study suffers from few limitations. Due to lack of funding, we were restricted to the urban areas of two provinces of Pakistan, and we were unable to survey the remote areas. There may be a bias at the providers' end because practitioners were reluctant or did not disclose the secrets of their formulae regarding actual active herb or combination. There may be a researcher bias as well, and patient may have not been telling the truth for the fear. However, the direct communication and explanation of the study purpose was served to minimize this bias.

Based on our findings, we recommend that the regulation of the CAM practices by a central authority must be regulated, putting more focus at the district level and primary care level to share and ensure communication around evidence based effective treatments for CAM users and providers. Public health awareness campaigns should broaden their scope involving print and social media to disseminate information for the CAM users and practitioners.

There is a lack of scientific evidence of CAM products use in hepatitis C. Prior assessment of these products should be performed for safety and efficacy, before they are used messily in human beings. It should be considered for future research so that these could be associated with HCV cure in patients. Referral by other hepatitis C patients indicates a satisfaction level around CAM and future research could explore their perceived satisfaction of these remedies in these patients.

## 5. Conclusions

This study has found that there exists a significant contribution of patients' beliefs on CAM, high costs of allopathic medicines, and social influences in hepatitis C patients' use of CAM. There is a need to support patients in optimizing modern treatment of hepatitis C, to facilitate greater access to hepatitis C treatment by alleviating cost factors and facilitating public awareness about effective treatments in Hepatitis C in order to promote evidence based medicine practices in curbing the high prevalence of the disease in Pakistan.

## Figures and Tables

**Table 1:** Demography and characteristics of respondents.

		Frequency	Percent
*Patients (n* *=* *284)*			
Age (years)	18–40	49	17.3
	41–60	86	30.3
	>60^*∗*^	149	52.5

Weight	40–60 kg	82	28.9
	61–80 kg^*∗*^	148	52.1
	81–100 kg	54	19.0

Gender	Female^*∗*^	173	60.9
	Male	111	39.1

Socioeconomic status (Tx. affording source)	Self	45	15.8
	Spouse	69	24.3
	Father/guardian^*∗*^	84	29.6
	Donations/Zakat/SS	16	5.7
	Hospital/Govt.	70	24.6

Perceived mode of acquiring infection	Blood transfusion	63	22.2
	Razor cuts/abrasions	89	31.3
	IVDU	14	4.9
	Dermal contact	25	8.8
	Unsterilized syringes	3	1.1
	Not known	90	31.7

Final diagnosis	Hepatitis C	156	54.9
	HCV + DM	61	21.5
	HCV + HTN	40	14.1
	HCV + CKD	27	9.5

Under CAM Tx since	1–3 months	170	59.9
	4–6 months	114	40.1

Allopathic Tx used	1-2 months	103	36.3
	3-4 months	76	26.7
	5-6 months	105	37.0

*CAM practitioners (n* *=* *133)*			
Speciality	Herbal	48	36.1
	Homeopathic	45	33.8
	Unani	34	25.6
	Spiritual healer	6	4.5

Formal education	Secondary or below	46	34.6
	Higher secondary	49	36.8
	Graduation or above	38	28.6

Experience	1–5 years	43	32.3
	6–10 years	15	11.3
	11–15 years	34	25.6
	16–20 years	41	30.8

Modal number of patients treated	21–50	48	36.1
	51–100	31	23.3
	101–200	30	22.6
	201–300	7	5.3
	501–1000	15	11.3
	Greater than 1000	2	1.5

Abbreviations: Tx = treatment, SS = social security, Govt = government, IVDU = intravenous drug user, HCV = hepatitis C, DM = diabetes mellitus, HTN = hypertension, and CKD = chronic kidney disease. ^*∗*^A *P* value less than 0.05 was considered significant.

**Table 2:** Perception of interviewed patients and providers regarding prevailing side effects and complications from allopathic medicines.

Prior medicine	Patient (*n* = 284)				Practitioner (*n* = 133)			
	SOF (*n* = 46)	PEG-INF (*n* = 122)	Silymarin (*n* = 47)	Other (*n* = 69)	SOF (*n* = 24)	PEG-INF (*n* = 100)	Silymarin (*n* = 2)	Other (*n* = 7)
Side effects								
Nausea/Vomiting	13 (28.3)	43 (35.2)	20 (42.6)	28 (40.6)	5 (20.8)	34 (34.0)	0 (0.0)	3 (42.8)
Skin allergy	4 (8.7)	24 (19.7)	2 (4.3)	11 (15.9)	0 (0.0)	2 (2.0)	1 (50.0)	1 (14.3)
Persistent fever	7 (15.2)	13 (10.6)	7 (14.9)	10 (14.5)	7 (29.2)	11 (11.0)	1 (50.0)	0 (0.0)
GIT disturbance	1 (2.2)	6 (4.9)	0 (0.0)	3 (4.3)	2 (8.3)	8 (8.0)	0 (0.0)	0 (0.0)
Multiple^*∗*^	21 (45.6)	36 (29.5)	18 (38.3)	17 (24.6)	10 (41.7)	45 (45.0)	0 (0.0)	3 (42.8)

Complications								
Anemia	9 (19.6)	43 (35.2)	11 (23.4)	13 (18.8)	7 (29.2)	30 (30.0)	0 (0.0)	3 (42.8)
Ascites	2 (4.3)	7 (5.7)	1 (2.1)	3 (4.3)	10 (41.7)	39 (39.0)	1 (50.0)	1 (14.3)
Constipation	11 (23.9)	21 (17.2)	17 (36.2)	25 (36.2)	2 (8.3)	15 (15.0)	0 (0.0)	2 (28.6)
Depression	10 (21.7)	14 (11.5)	4 (8.5)	5 (7.2)	3 (12.5)	9 (9.0)	1 (50.0)	0 (0.0)
Kidney problem	2 (4.4)	5 (4.1)	5 (10.6)	1 (1.4)	1 (4.2)	6 (6.0)	0 (0.0)	0 (0.0)
Multiple^*∗*^	12 (26.1)	32 (26.2)	9 (19.1)	22 (31.9)	1 (4.2)	1 (1.0)	0 (0.0)	1 (14.3)

Abbreviations: SOF = sofosbuvir, PEG-INF = pegylated-interferon, GIT = gastrointestinal tract, and ^*∗*^multiple = more than one symptom.

**Table 3:** Comparison of participants' perspectives regarding referral and shift to CAM use for the treatment of hepatitis C.

Parameter	Subcategory	Patient (*n* = 284)		Mean rank	*P* value^*∗*^	Practitioner (*n* = 133)			Mean rank	*P* value^*∗*^
		F (*n* = 207)	M (*n* = 77)			Herbal (*n* = 48)	Homeo. (*n* = 45)	Unani, spiritual (*n* = 40)		
Referral source	Health professionals	39 (18.8)	14 (18.2)	147.9	0.219	3 (6.3)	0 (0.0)	0 (0.0)	22.5	0.026
	Relative or neighbours	110 (53.1)	50 (64.9)	156.8		7 (14.6)	8 (17.8)	10 (17.6)	77.3	
	Printed advertisement	11 (5.3)	1 (1.3)	115.8		10 (20.8)	2 (4.4)	6 (17.6)	69.5	
	By other patients	7 (3.4)	3 (3.9)	146.6		26 (54.2)	32 (71.1)	17 (47.2)	61.8	
	Care giver	40 (19.3)	9 (11.7)	130.1		2 (4.2)	3 (6.7)	7 (17.6)	85.3	

Reasons for patients to switch to CAM	Side effects	105 (50.7)	43 (55.8)	145.3	0.641	27 (56.3)	25 (55.6)	21 (44.1)	69.6	0.030
	Confidence in CAM	49 (23.7)	21 (27.3)	146.6		5 (10.4)	10 (22.2)	8 (23.5)	81.4	
	Expensive allopathic	43 (20.8)	11 (14.3)	132.9		10 (20.8)	4 (8.9)	8 (23.5)	55.1	
	Easy access	6 (2.9)	1 (1.3)	124.3		4 (8.3)	5 (11.1)	2 (5.9)	55.2	
	Not described	4 (1.9)	1 (1.3)	132.4		2 (4.2)	1 (2.2)	1 (2.9)	34.0	

Abbreviations: CAM = complementary and alternative medicines, F = females, M = males, homeo. = homeopathic, and ^*∗*^Chi-Square test; *P* value less than 0.05 was considered significant. Mean ranks of the variables are obtained by Kruskal–Wallis test.

## Data Availability

The datasets supporting the conclusions of this article are available from the corresponding author upon request (tofeeq.urrehman@qau.edu.pk).

## Abbreviations

CAM: Complementary and alternative medicines
DDAs: Direct-acting antivirals
HCV: Hepatitis C virus
LMICs: Lower-middle-income countries
NCCAM: National Center for Complementary and Alternative Medicine
PEG-INF: Pegylated-interferon.
